# Psychological Distress following Injury in a Large Cohort of Thai Adults

**DOI:** 10.1371/journal.pone.0164767

**Published:** 2016-10-24

**Authors:** Thanh Tam Tran, Joel Adams-Bedford, Vasoontara Yiengprugsawan, Sam-Ang Seubsman, Adrian Sleigh

**Affiliations:** 1 Department of Global Health and National Centre for Epidemiology and Population Health, Research School of Population Health, ANU College of Medicine, Biology and Environment, The Australian National University, Canberra, Australia; 2 The Australian National University Medical School, Canberra, Australia; 3 Centre for Research on Ageing, Health and Wellbeing, Research School of Population Health, ANU College of Medicine, Biology and Environment, The Australian National University, Canberra, Australia; 4 School of Human Ecology, Sukhothai Thammathirat Open University, Nonthaburi, Thailand; University of Alabama at Birmingham, UNITED STATES

## Abstract

**Introduction:**

Injury and psychological distress are public health priorities because of their high occurrence in the population. This study examines the longitudinal effects of injury characteristics on psychological distress.

**Methods:**

Study participants were enrolled distance learning Thai adults (N = 42,785 at 2013 follow-up) residing nationwide. We analysed 2009 and 2013 data. Injury questions included injury prevalence, causes and levels of severity. Distress was measured using the standard Kessler-6. To assess the risk for post-injury distress, we used multinomial logistic regression investigating psychological distress in 2013 as an outcome including injury categories in both 2009 and 2013 as predictors, adjusted for sociodemographic factors.

**Results:**

Overall injury was predictive of psychological distress. Both types of injury (traffic and non- traffic) associated with increasing psychological distress. Those that had experienced both types of injuries in the previous year had higher odds of developing psychological distress compared to those who experienced just one type. In 2013, adjusted psychological distress odds ratios were 1.46 [95% Confidence Interval 1.14–1.87] for traffic injury only; 1.26 [1.13–1.40] for non-traffic injury only; and 2.71 [2.19–3.35] for both traffic and non-traffic injuries. Increasing frequency of injury and increasing injury severity were also linked to elevated psychological distress among our Thai cohort members.

**Conclusions:**

Our results revealed a significantly high risk of psychological distress following injury. With increasing occurrence of injury, especially traffic injuries in low and middle income countries such as Thailand, future policies should not only focus on physical care but also address psychological distress as an important consequence of injury.

## Introduction

Globally, injury and psychiatric disorders are responsible for a substantial disease burden and related premature mortality. Together, they account for 18% of the global loss of Disability-Adjusted Life Years (DALYs) [1]. Injuries have been a public health priority because of their burden and associated mortality [2]. They are especially important in low and middle income countries, which account for 90% of the global burden [3]. Injury often leads to reduced quality of life, impaired physical and social functioning, and substantial pain and suffering [4–6]. Other results from the Thai cohort study found that injury status was linked to both physical and mental health [7]. A Danish cohort study found that 7.3% reported having long-term effects from injuries [8]. In other cohort studies, injuries have also been connected to significant long-term deficits in quality of life [9, 10].

An association between injury and psychological distress does not of itself establish the direction of any causal links [11]. One major question is the mechanism, which connects psychological distress and injury and whether the mechanisms vary for different injury categories. It should be noted that psychological distress combines a number of anxiety and mood disorders [12]. Injury is also a broad category that incorporates the results of physical or chemical trauma in various settings (home, work, road, sport) with a variety of circumstances related to intent and agency.

In Thailand, a health-risk transition is underway. As the country embraces globalisation and modernisation, the health of the Thai population is moving from traditional challenges with infectious diseases and premature maternal-child mortality to emerging chronic conditions and injury. Between 1990 and 2010, the number of injury deaths in the country has increased by almost 50% [13]. By 2013, injury had overtaken communicable diseases as the country’s second leading cause of Disability-Adjusted Life Years lost. Almost 40% of injury deaths were road traffic related. Road injury was also responsible for 5.8% of total DALYs in 2013, second to ischaemic heart disease [13].

In our Thai cohort study, two injury categories were investigated—traffic and non-traffic injury. We hypothesised that injury could result in higher levels of psychological distress and tested the effect of injury on psychological distress over time, at one and four years.

## Methods

### Study population and data collection

We used data from the Thai Cohort Study (TCS), a large-scale cohort research project investigating health risks and outcomes among Thai adults. The cohort members were 87,151 distance learning students enrolled at Sukhothai Thammathirat Open University (STOU) in 2005. They lived nationwide embedded in their communities and were unable to attend on-campus due to personal and professional commitments as well as modest socioeconomic means. They were aged up to 87 years at baseline in 2005 when they completed a mailed questionnaire, but almost all were aged 20–50 years.

This cohort shares many common features with the general Thai population including male-female proportion, ethnicity, religion, income, and geographic residence. However, cohort members are better educated than the general Thai population and are more urbanized, thus, representing future trends in the Thai population [14–16]. The questionnaires gathered information on many sociodemographic attributes, health and disease status, well-being, social support, psychological status, recent injury, health related behaviour (smoking and drinking), transportation and family health history. In 2009 and 2013, two follow-up surveys were conducted (n = 60,569 in 2009 and n = 42,785 in 2013), with an attrition rate of approximately 30% at each wave, partly due to loss of mail contact related to students graduating or suspending their studies. This study used data from the 2009 and 2013 questionnaires to investigate the relationship between different types of injury and psychological distress. A total of 40,157 people answered the 2013 Kessler-6 questions and both the 2009 and 2013 injury questions, and thus made up the final study sample.

### Psychological distress

Psychological distress was measured from a standard set of questions known as the Kessler-6 scale. It assessed how much of the time during the past 4 weeks an individual felt “nervous”, “so sad nothing could cheer you up”, “restless or fidgety”, “hopeless”, “that everything was an effort” and “worthless”. Answers were coded as “all of the time”, “most of the time”, “some of the time”, “a little of the time”, or “none of the time”. To determine psychological distress scores, responses for each question were summed up, resulting in a possible range from 6 to 30. High scores on the Kessler are associated with a potential psychiatric disease (21). For analysis we converted Kessler scores into 3 categories: low distress (scores of 6–11), moderate distress (12–17) and high distress (18–30). This corresponds with international Kessler standard measures indicating that the high distress group is most likely to have a mental disorder [17].

### Injury

Two sets of questions dealt with injury–one for traffic and one for non-traffic injury in both 2009 and 2013. The initial question for both types of injuries were “In the last 12 months, how many times did you get injured in a traffic crash?” and “In the last 12 months, how many times did you have a non-traffic injury?”. The response categories for each question include:”never”,”one”,”two”,”three”, or”four or more”. Other descriptive information was collected about each injury, such as if an injury required medical care and limited activity for days following the incident. For traffic injuries, additional information was collected about the role the injured person played in the traffic incident. For non-traffic injury, we recorded information on the mechanism and place of injury.

### Covariates

The relationship between psychological distress and injury could be confounded by the influence of other factors. Accordingly, we gathered data on a wide array of demographic, socioeconomic and geographic attributes that were known to be potential confounders. Sociodemographic covariates recorded included age groups (4 categories: <30, 30–39, 40–49, and ≥50 years), sex (male or female), marital status (single or married/living with a partner), personal monthly income (4 categories: ≤7000, 7001–10,000, 10,001–20,000, 20,001–30,000, and >30,000 Baht), and residence (rural or urban).

Other covariates included for analysis were alcohol consumption, drink driving in the past 12 months, cigarette smoking, doctor-diagnosed chronic conditions, and body mass index (BMI). Those who reported zero alcohol drinks per week were considered non-drinkers; and those who reported one or more drinks per week were classified as drinkers. Drink driving was assessed by a dichotomized yes/no question: “During the last 12 months have you driven a motor vehicle after consuming 3 or more glasses of alcohol?” Participants were asked if they were current smokers, or have smoked before but now stopped completely (former smokers), or never smoked. Participants were also asked to report whether they have been told by a doctor that they have one of the following conditions: diabetes (needing insulin or not), high cholesterol, high blood pressure, ischemic heart disease, stroke, cancers (liver, lung, digestive system, breast or other), goiter/thyroid abnormality, epilepsy, liver disease, chronic kidney disease, depression/anxiety, arthritis, pneumonia, chronic bronchitis, asthma, malaria, dengue fever, tuberculosis, other chronic infection, or any other diseases. Responses for all the health conditions were summed and grouped into 3 categories by number of illnesses: 0, 1, or ≥2. BMI was reported in kg/m2, calculated as weight over height squared. Asian categories were used as follows: <18.5 (underweight), ≥18.5 to <23 (normal), ≥23 to <25 (overweight at risk), ≥25 to <30 (obese I), ≥30 (obese II) [18].

### Statistical analysis

As the injury question asked for information in the past year while psychological distress questions assessed the past four weeks, it is therefore reasonable to assume that injury in 2013 precedes psychological distress in 2013. Our analysis investigated the effects of injury on psychology distress at four-year follow-up (2009–2013).

First, baseline (2009) demographic characteristics of participants at different psychological distress levels (in 2013) and injury status (in 2009 and 2013) were compared using bivariate frequency distribution. For each category, a chi-square test was conducted to observe differences between groups. Characteristics of injuries observed in our cohorts for both years were also described with descriptive frequency distributions.

To study the effects of different types of injury on psychological distress, we performed a set of five multinomial logistic regressions with different injury categories in both 2009 and 2013 as predictors, and psychological distress in 2013 as multinomial outcome (low, moderate, and high), adjusting for baseline sociodemographic factors mentioned previously. The predictors for the five regressions were: (1) total number of injuries derived from a sum of both types of injury reported; (2) categories of injuries (traffic only, non-traffic only, or both); (3) injury severity: serious injury if participants answered “Yes” to the question “Did this injury limit your activities for one day or more?” and mild injury if answered “No”; (4) reported number of traffic injuries stratified by injury severity; and (5) reported number of non-traffic injuries stratified by severity. Participants with missing information on any one of the potential confounders were dropped from the final logistic regression analyses, thus the sample size for the logistic regression was smaller than our initial sample size.

### Data processing

Questionnaire responses were digitised by optical scanning and subsequently edited using Thai Scandevet, SQL and Statistical Package for the Social Sciences (SPSS). For statistical analysis we used Stata/SE 13.1.

### Ethical considerations

Informed written consent was obtained from all participants. Ethics approval was obtained from Sukhothai Thammathirat Open University Research and Development Institute (protocol 0522/10) and the Australian National University Human Research Ethics Committee (protocols 2004/344 and 2009/570).

## Results

### Characteristics of TCS members by injury and psychological distress (2009)

Frequencies of the main covariates of the cohort stratified by psychological distress status are presented in Table 1. Younger age, being female, and being single was more frequent in individuals with high psychological distress. High income was linked to a lower likelihood of reporting moderate to high psychological distress. Risk-taking behaviours including cigarette smoking, alcohol drinking and drink driving were more frequent in individuals with high psychological distress. For BMI, a U-shaped relationship was observed with the highest prevalence of high distress in underweight and in obesity class II group. More than one third of individuals with a high level of distress in 2009 also reported high distress in 2013.

**Table 1:** Demographic and behavioural characteristics of Thai Cohort Study members in 2009, stratified by level of psychological distress in 2013 (N = 40,157).

Demographic attributes		Low distress		Moderate distress		High distress	
		N	%	N	%	N	%
**Age groups (years)**							
	<30	4,785	49.4	4,001	41.3	905	9.3
	30–39	9,513	55.2	6,430	37.3	1,299	7.5
	40–49	6,426	62.5	3,285	31.9	577	5.6
	≥50	1,962	66.8	839	28.6	135	4.6
**Sex**							
	Male	10,374	57.5	6,482	35.9	1,177	6.5
	Female	12,312	55.6	8,073	36.5	1,739	7.9
**Marital status**							
	Single	7,664	53	5,583	38.6	1,223	8.5
	Partnered	13,536	58.9	7,964	34.7	1,471	6.4
**Personal monthly income†**							
	≤7,000	3,287	48.4	2,778	40.9	721	10.6
	7,001–10,000	4,408	52.5	3,319	39.5	674	8
	10,001–20,000	8,001	56.7	5,131	36.4	967	6.9
	20,001–30,000	3,769	64.6	1,771	30.4	295	5.1
	>30,000	2,601	66.8	1,137	29.2	158	4.1
**Residential status^**							
	Rural	10,011	56.1	6,525	36.6	1,312	7.4
	Urban	12,190	56.9	7,726	36	1,517	7.1
**Smoking status**							
	Current smoker	1,590	51.7	1,221	39.7	267	8.7
	Former smoker	3,229	57.2	2,008	35.6	404	7.2
	Never smoked	17,613	57	11,112	35.9	2,188	7.1
**Alcohol drinking**							
	No	16,231	57.6	9,978	35.4	1,972	7
	Yes	5,632	54	3,976	38.1	815	7.8
**Driving after drinking in the previous 12 months**							
	Yes	4,366	52.5	3,288	39.5	660	7.9
	No	15,843	57.9	9,636	35.2	1,870	6.8
	Do not drive	1,795	56.3	1,132	35.5	260	8.2
**Doctor diagnose chronic conditions**							
	0	12,207	58	7,452	35.4	1,402	6.7
	1	7,276	55.8	4,816	36.9	947	7.3
	≥2	3,147	53.1	2,238	37.8	539	9.1
**Body Mass Index**							
	Underweight	1,851	52.3	1,401	39.6	289	8.2
	Normal	10,921	56.3	7,081	36.5	1,395	7.2
	Overweight	4,357	58.1	2,667	35.6	476	6.3
	Obese I	4,310	58.2	2,561	34.6	534	7.2
	Obese II	823	53.7	560	36.5	151	9.8
**Psychological distress in 09**							
	Low distress	14,142	64	7,385	33.4	555	2.5
	Moderate distress	4,039	28.5	8,525	60.2	1,606	11.3
	High distress	345	12.2	1,402	49.6	1,082	38.2
**Injury in 2009**							
	No injury	14,405	60	8,095	33.7	1,496	6.2
	Injury	8,281	51.2	6,460	40	1,420	8.8
**Injury in 2013**							
	No injury	14,329	60.2	8,005	33.6	1,463	6.1
	Injury	8,357	51.1	6,550	40	1,453	8.9

† in Thai baht (in July 2009, 1 baht ≈ 0.0292 USD)

^ All associations were statistically significant (p<0.05) except for residential status.

Those that were previously injured were more likely to report psychological distress compared to those who did not report an injury in 2009–8.8% of people injured in 2009 reported high distress in 2013, compared to 6.2% of those with no injury. A similar scenario was observed in 2013, whereby 8.9% of people reported injury (vs 6.1% of uninjured people).

### Injury frequency, mechanisms and location in 2009 and 2013

Injury characteristics among TCS members in 2009 and 2013 were very similar (Table 2). The proportions of those incurring an injury were 37.6% in 2009 and 38.8% in 2013. Roughly 8 to 9 percent of participants reported having at least one incident of traffic injury and 35% reported having at least one incident of other types of injuries (e.g fall, assault). Approximately two-fifths of traffic injured compared to one-fifth of non-traffic injuries people received medical care; 43% to 44% of people with traffic injuries and 22% to 23% of people with non-traffic injuries reported limited activities for at least one day. The majority of traffic injured participants were drivers at the time of the incident, 16% to 19% were passengers, and 4% were pedestrians. The three most common types of non-traffic injuries were stab/cut, blunt force other than assault, and fall. Most of those injuries were unintentional accidents (>95%). The majority of non-traffic injury happened at home, followed by non-agricultural workplaces.

**Table 2:** Types of injuries, mechanisms and locations in the Thai cohort (2009 and 2013) (N = 40,157).

Injury Attributes		2009		2013	
		N	%	N	%
**All injuries**					
	None	23,996	62.3	23,797	60.87
	One	5,339	13.9	5,787	14.8
	Two	3,378	8.8	4,254	10.88
	Three or more	5,817	15.1	5,254	13.44
**Traffic injury**					
	None	35,727	90.8	36,685	92.27
	One	2,885	7.3	2,401	6.04
	Multiple	731	1.9	673	1.69
	Medical care received (yes)	1,456	40.5	1,284	42.04
	Limit activities (yes) *	1,564	43.6	1,350	44.1
**Role during incident**					
	Driver	2,704	76.3	2,370	78.55
	Passenger	672	19	507	16.8
	Pedestrian	167	4.7	140	4.64
**Non-traffic injuries**					
	None	25,567	65	25,297	64.06
	One	5,104	13	5,390	13.65
	Multiple	8,673	22	8,803	22.29
	Medical care received (yes)	3,124	23.1	3,159	22.47
	Limit activities (yes) *	3,465	25.6	3,424	24.36
**Mechanism of non-traffic injury**					
	Assault	150	1.3	113	0.93
	Other blunt force	2,560	22.3	2,432	19.96
	Gun shot	9	0.1	6	0.05
	Stab/Cut	2,561	22.4	2,807	23.03
	Fall	2,298	20.1	2,292	18.81
	Thermal	406	3.5	371	3.04
	Poison	56	0.5	50	0.41
	Bite/Sting	1,037	9.1	1,111	9.12
	Choking	24	0.2	23	0.19
	Other	2,357	20.6	2981	24.46
**Intentionality of non-traffic injury**					
	Unintentional accident	12,800	96.6	13,358	95.73
	Intentional by another person	230	1.7	198	1.42
	Intentional no other person involved	221	1.7	398	2.85
**Location of non-traffic injury occurrence**					
	Home	5,630	43.8	6,381	47.17
	Sport	1,433	11.2	1,202	8.88
	Workplace- agricultural	1,287	10.1	1,536	11.35
	Workplace- non-agricultural	2,903	22.7	2,986	22.07
	Other	1,557	12.2	1,424	10.53

* The participants were asked to report whether the injury limited their normal activity for one day or more

### Injury as predictor of psychological distress

To investigate the effects of different injury types on psychological distress, we performed a set of five multinomial logistic regressions with different types of injuries in both 2009 and 2013 as predictors and 2013 levels of psychological distress as outcomes (low vs high and low vs moderate), controlled for 2009 age groups, sex, marital status, income, smoking, alcohol drinking, drink driving, co-morbidity, BMI, and psychological distress. The results are reported in Table 3.

**Table 3:** Adjusted association between different injury categories and psychological distress, using multinomial logistic regression analysis.

Injury categories		Moderate vs Low Distress		High vs Low Distress	
		OR	95% CI	OR	95% CI
*Total number of injury *					
	**2009**				
	0	1.00		1.00	
	1	1.03	0.95 ─ 1.11	1.09	0.94 ─ 1.25
	2	1.03	0.94 ─ 1.13	1.02	0.86 ─ 1.22
	3 or more	1.05	0.98 ─ 1.14	1.10	0.96 ─ 1.26
	**2013**				
	0	1.00		1.00	
	1	1.06	0.98 ─ 1.14	1.06	0.92 ─ 1.23
	2	**1.34**	1.23 ─ 1.45	**1.38**	1.18 ─ 1.62
	3 or more	**1.50**	1.38 ─ 1.62	**1.83**	1.60 ─ 2.11
*Causes of injury*		* *	* *	* *	* *
	**2009**				
	No injury	1.00		1.00	
	Traffic only	**1.18**	1.03 ─ 1.36	**1.31**	1.03 ─ 1.68
	Non-traffic only	1.03	0.97 ─ 1.10	1.07	0.96 ─ 1.20
	Both	1.06	0.94 ─ 1.20	1.12	0.91 ─ 1.37
	**2013**				
	No injury	1.00		1.00	
	Traffic only	1.11	0.97 ─ 1.28	**1.46**	1.14 ─ 1.87
	Non-traffic only	**1.22**	1.16 ─ 1.30	**1.26**	1.13 ─ 1.40
	Both	**1.97**	1.72 ─ 2.25	**2.71**	2.19 ─ 3.35
*Injury severity*		* *	* *	* *	* *
	**2009**				
	No injury	1.00		1.00	
	Mild injury	1.03	0.97 ─ 1.10	1.10	0.98 ─ 1.23
	Serious injury	**1.15**	1.05 ─ 1.25	**1.20**	1.04 ─ 1.40
	**2013**				
	No injury	1.00		1.00	
	Mild injury	**1.23**	1.16 ─ 1.31	**1.23**	1.10 ─ 1.39
	Serious injury	**1.37**	1.25 ─ 1.49	**1.77**	1.53 ─ 2.06
*Traffic injury by injury severity *					
	**2009**				
	No injury	1.00		1.00	
	Mild injury	**1.17**	1.04 ─ 1.31	1.13	0.93 ─ 1.38
	Serious injury	1.14	1.00 ─ 1.30	**1.35**	1.09 ─ 1.66
	**2013**				
	No injury	1.00		1.00	
	Mild injury	**1.28**	1.13 ─ 1.45	**1.65**	1.35 ─ 2.03
	Serious injury	**1.52**	1.32 ─ 1.76	**2.11**	1.68 ─ 2.64
*Non-traffic injury by injury severity *					
	**2009**				
	No injury	1.00		1.00	
	Mild injury	1.02	0.95 ─ 1.07	1.06	0.94 ─ 1.19
	Serious injury	**1.14**	1.04 ─ 1.25	1.17	0.99 ─ 1.37
	**2013**				
	No injury	1.00		1.00	
	Mild injury	**1.26**	1.19 ─ 1.34	**1.23**	1.09 ─ 1.37
	Serious injury	**1.33**	1.21 ─ 1.46	**1.76**	1.51 ─ 2.06

Bold values are statistically significant (p<0.05)

Regressions adjusted for age, sex, marital status, personal income, smoking, alcohol drinking, drink driving, number of chronic health condition, and body mass index

Increasing number of injuries was associated with elevated levels of psychological distress. This effect however, was only observed up to one year post-injury (in 2013) (OR 1.83, 95% CI 1.60–2.11 in high vs. low distress with 3 or more injuries compared to none) but not four years (for injuries in 2009).

With regard to causes of injury, four years post-incident, the negative effect of traffic injury (but not non-traffic or both types) on participants’ psychological wellbeing could still be observed (OR for moderate distress vs low distress = 1.18, 95% CI 1.03–1.36; OR high distress vs low distress = 1.31, 95% CI 1.03–1.68, respectively). Considering the more recent event in 2013 (one year post-incident), the odds were even higher. Furthermore, the damages were compounded for those that experienced both type of injuries and hence reported the highest odds of having moderate or high psychological distress.

For both, traffic and non-traffic injuries increasing injury severity was linked to increasing psychological distress. In general, compared to those with no injury, people who experienced serious injury in 2009 were 1.15 times more likely to report moderate distress and 1.20 times more likely to report high distress as opposed to reporting low distress. Notably, the effect of the injuries that were experienced more recently (in 2013) were stronger.

Even those with mild injury were also significantly more likely to report worse distress. Cohort members with serious injury were 1.37 and 1.77 times more likely to report moderate or high distress. Similar patterns could be observed when considering traffic and non-traffic injuries separately.

## Discussion

Our findings in a large cohort of Thai adults revealed that injuries could lead to psychological distress. However, the effect of injury on distress attenuated over time. The experience of either type of injury–traffic or non-traffic–were associated with subsequent psychological distress; and for those who experienced both types, the effects were compounded. Both the increase in number of injuries and in injury severity were linked to elevated levels of distress. Such effects could be observed when considering traffic and non-traffic injuries separately or together.

A substantial amount of literature has established the relationship between injury and post-injury distress [7, 8, 19, 20]. For example, Wrengera *et al*. demonstrated that 40% of injury patients would qualify for a psychological diagnosis [21]. Although not examined in our study, a number of different psychological and psychiatric changes have been reported after injuries including depression [22], anxiety [23], substance abuse [24], and post-traumatic stress [25].

Previously, in the Thai Cohort Study, we have shown that injury led to worse physical and mental wellbeing [7]. This paper complements and builds on previous findings by investigating different injury categories and further investigating their effects across time. A cohort study by Toft, Moller and Laursen found that those with injuries reported poor health and self-reported depression even up to 10 years after the incident [26]. In this current study, the effect of injury on distress was lost just four years post injury, except for those with more serious injury, such as traffic injuries. This difference could be due to the fact that injuries reported in our study were in general fairly mild, while, Toft and colleagues recruited participants that were seriously injured and admitted to hospitals [26].

Despite a large body of literature on injury and psychological distress, few studies have attempted to compare differences between traffic and non-traffic injuries. The fact that we found increased distress after traffic injury is no surprise as there has been well-established literature on this subject, especially in terms of post-traumatic stress disorder [27, 28]. However, for non-traffic injuries, the evidence was scarce and conflicting. Considering burns, for example, Sareen et al. reviewed the literature for risk factors of post-injury mental health problems and found that the majority of studies have not established a relationship for either injury severity or total body surface area [4]. As most studies that found associations between injury and post-injury distress among clinical trauma patients that tended to be more serious in nature, hence, having a higher likelihood of adverse impacts to the patients’ mental health [6, 29]. In contrast, the injury profile in our cohort was relatively minor, most was unintentional with high proportions of cuts and falls. Nevertheless, in this cohort, we could still observe post-injury distress, and in some cases the effect of more serious injury could be observed up to four/five years after the incident.

The advantage of the present study is the large sample of Thai adults. The data derived from a comprehensive questionnaire that allowed for the examination of a number of covariates, including socioeconomic, environmental, social and geographic variables, permitting a more thorough investigation of factors that could influence the injury and distress effects. Another advantage is that the questionnaire asked about different types of injuries, which allows us to examine the relationship with psychological distress according to injury types. Further, the cohort is being examined at a number of data points, which will facilitate future longitudinal analyses to better understand causality, particularly given the potential for bi-directional relationships between injury and psychological distress.

One limitation of the present study was the inability to characterize long-term psychological distress status because distress was only asked for the previous four weeks. Injury questions were directed at the preceding twelve months from the time that questionnaire was mailed out. This makes it difficult to assess causality between both variables. As well, it was not possible to use 2005 data because the injury question and the psychological distress questions were different. The injury question in 2005 was asked only regarding the most serious injury and the Kessler 6 distress was not included. As such it was not possible to compare 2005 data with 2009 and 2013.

Our research provides evidence that psychological distress is an important consequence of injury. With increasing prevalence of injuries, especially traffic injuries in lower-middle income countries such as Thailand, this information is important in injury care and management, indicating the potential need for increasing use of post-injury mental health services. To minimize the burden of injury, future policies should not just focus on physical care but also address distress as an important consequence and ensure access to psychological care. As well, more studies are needed to further characterize the injury-distress relationship to determine appropriate interventions.
